# New Appliances and Things Medical

**Published:** 1900-07-21

**Authors:** 


### NEW APPLIANCES AND THINGS MEDICAL.

**[We shall be glad to receive, at our Office, 28 & 29, Southampton Street, Strand, London, W.C., from the manufacturers, specimens of all new preparations and appliances which may be brought out from time to time.]**

### EUREKA MALTED FOOD.

(Coombs' Eureka Aerated Flour Company, Limited, Lenton, Boulevard, Nottingham.)

This food belongs to the popular class of semi-predigested cereal flours. If mixed with milk and allowed to stand for a few minutes, the malt ferment present in the powder rapidly converts the starch into soluble carbohydrates or sugars. For those who are unable to digest the unaltered flour, this process of predigestion has obvious advantages.

### PARASITINE AND PARASITINE OINTMENT.

(Wednesfield Chemical Syndicate, Limited, Wednesfield, Wolverhampton.)

These preparations would appear to possess powerful germicide properties; and whether in the fluid form or when combined in the form of an ointment, exercise curative powers on those skin affections which are due to living parasites. For instance, in eczema, ringworm, and scabies, if well rubbed into the skin, a marked and distinct improvement in the condition may be expected.

### CLOUDY AMMONIA AND ANTISEPTIC SOAP.

(Scrubb and Co., Guildford Street, Lambeth, London, S.E.)

Scrubbs' cloudy ammonia is now considered such a household necessity that it needs no further description. The antiseptic soap which is being supplied by the same firm, and is intended for use in conjunction with the cloudy ammonia, requires a few words of explanation. This soap is so prepared that it contains no free alkali; when such an alkali is desired it can be at once obtained by adding a few drops of cloudy ammonia to the water. The advantages of this combination, over soaps, which contain in themselves free alkalis of the nature of soda, are very apparent. Ammonia is refreshing and cleansing to the skin, and has no destructive influence on the delicate epithelium, as is the case where strong sodas are employed; and moreover, owing to its rapid powers of evaporation, having done its work, it is immediately dissipated into space, leaving the skin invigorated and purified. Scrubbs' cloudy ammonia and antiseptic soap should always be used when the water is hard, and on other occasions whenever possible.

### SOUTH AUSTRALIAN WINES (ORION BRAND).

(E. Burney Young, 35, Walbrook, London, E.C.)

We have examined a number of samples of South Australian wines belonging to the Orion series, which appear to us to reflect the greatest credit on the wine-producing powers of South Australia. They are all perfectly pure, exceedingly pleasant in flavour, and although perhaps richer than we are accustomed to in the European wines of corresponding type, it is a fault easily corrected by the addition of water. The list of wines which we have examined includes a fine rich Burgundy, red wines of lighter properties called Ruby and Cabernet respectively, and a fourth which is a good plain dinner claret. Among the white wines we would call attention to the Riesling, a most delicious wine especially suitable for dilution with aërated waters, and particularly to be recommended in hot weather. The Orion brandy is a spirit distilled from the grapes of South Australia. Although it can hardly be compared to a genuine Cognac it is very superior to what one usually obtains under the name of brandy. It is perfectly pure, and for this reason has obvious advantage either for invalid or general use.

